# Environmental Enrichment Protects against Neurotoxic Effects of Lipopolysaccharide: A Comprehensive Overview

**DOI:** 10.3390/ijms24065404

**Published:** 2023-03-11

**Authors:** Eugenia Landolfo, Debora Cutuli, Davide Decandia, Francesca Balsamo, Laura Petrosini, Francesca Gelfo

**Affiliations:** 1 IRCCS Fondazione Santa Lucia, Via Ardeatina 306, 00179 Rome, Italy; 2 Department of Psychology, Sapienza University of Rome, Via dei Marsi 78, 00185 Rome, Italy; 3 Department of Human Sciences, Guglielmo Marconi University, Via Plinio 44, 00193 Rome, Italy

**Keywords:** neurotoxicity, lipopolysaccharide, environmental enrichment, animal models, rodents, neuroinflammation, maternal immune activation

## Abstract

Neuroinflammation is a pathophysiological condition associated with damage to the nervous system. Maternal immune activation and early immune activation have adverse effects on the development of the nervous system and cognitive functions. Neuroinflammation during adulthood leads to neurodegenerative diseases. Lipopolysaccharide (LPS) is used in preclinical research to mimic neurotoxic effects leading to systemic inflammation. Environmental enrichment (EE) has been reported to cause a wide range of beneficial changes in the brain. Based on the above, the purpose of the present review is to describe the effects of exposure to EE paradigms in counteracting LPS-induced neuroinflammation throughout the lifespan. Up to October 2022, a methodical search of studies in the literature, using the PubMed and Scopus databases, was performed, focusing on exposure to LPS, as an inflammatory mediator, and to EE paradigms in preclinical murine models. On the basis of the inclusion criteria, 22 articles were considered and analyzed in the present review. EE exerts sex- and age-dependent neuroprotective and therapeutic effects in animals exposed to the neurotoxic action of LPS. EE’s beneficial effects are present throughout the various ages of life. A healthy lifestyle and stimulating environments are essential to counteract the damages induced by neurotoxic exposure to LPS.

## 1. Introduction

Neurotoxicity is a term that refers to the direct or indirect effects of any potentially harmful substance affecting the nervous system of humans or animals [1,2]. Numerous substances, natural or artificial, can produce human neurotoxic diseases, and many others are used as experimental tools to disturb or damage the nervous system in animal models [1,3]. Some of these neurotoxins act directly on neural cells [4], while others alter metabolic processes that are strongly dependent on the neural system. Symptoms can appear immediately after the exposure or be delayed [2]. The nervous system responds with multiple functional reactions to the exposure to neurotoxic substances, and these responses give rise to a series of neurological and psychiatric phenomena, many of which typify the clinical manifestations of diseases of nontoxic origin [1].

### 1.1. Lipopolysaccharide (LPS)

The bacterial cell surface is the first line of defense between the bacterium and the world. The structure and composition of the bacterial cell surface can determine the immune system responses and the outcomes of bacteriophage infections [5]. One of the most studied bacterial surface molecules is the glycolipid known as lipopolysaccharide (LPS), which is produced by most Gram-negative bacteria [6] and is present in different forms with respect to the type of bacterium. LPS’s function is to stabilize the cell membrane architecture in bacteria.

In humans, the principal derivation of LPS are gastrointestinal tract-resident facultative anaerobic Gram-negative bacilli, among them *Bacteroides fragilis* and *Escherichia coli*. However, LPS’s presence is generally interpreted as toxic and triggers damage signals and mechanisms that underlie the innate immune response and inflammation [7,8]. LPS is also referred to as an endotoxin because it is an integral part of the cell [9]. Located in the outer flap of the outer membrane of the bacterial cell, LPS is involved in the interaction of the cell with the environment [10].

**Figure 1:**
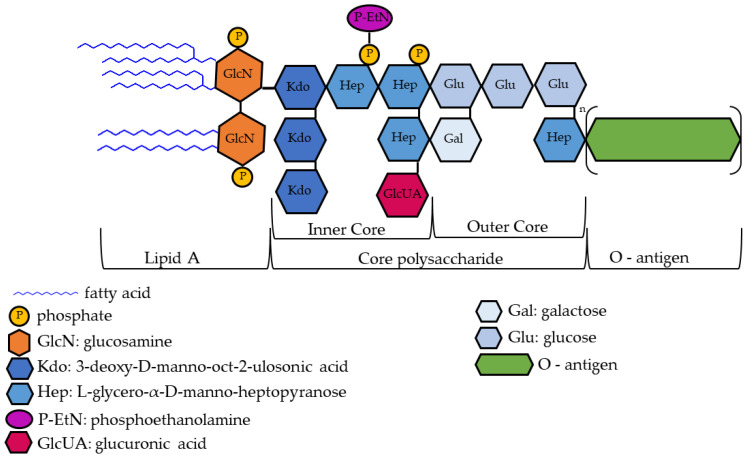
Exemplary drawing of lipopolysaccharide (LPS) structure. For details on LPS structure and regulatory steps in its biosynthesis, see [13].

Although complex, the chemical structure of some forms of LPS, in particular, that of Enterobacteriaceae (*Escherichia coli*, *Shigella,* and *Salmonella*), is well known. LPS consists of a hydrophobic membrane anchor portion known as lipid A, and a nonrepeating core oligosaccharide coupled to a distal polysaccharide, O-antigen, that extends from the bacterial surface [9,11]. The lipid component A varies from one organism to another and is essential for conferring specific pathogenic attributes to bacteria [12]. The lipid section that represents the principal endotoxin is the hydrophobic portion and consists of an acylated and 1,4′ bisphosphorylated β (1 → 6)-linked glucosamine (GlcN) disaccharide [13]. The architecture of the core oligosaccharide is relatively preserved within a species; structures can vary widely between species in terms of number and kind of sugars, extent of branching, and occurrence of non-glycosyl substituents [14,15]. In *Escherichia coli*, the core polysaccharide can be subdivided into two cores, namely, the inner and outer cores. The inner core usually contains a conserved structural element of 3-deoxy-α-D-manno-oct-2-ulopyranosonic acid (Kdo), L-glycero-α-D-manno-heptopyranose (Hep), and phosphate residues [13]. O-antigens are the most variable part of the LPS molecule; changes in the O-antigen plausibly play an essential role in the infection process. The O-antigen mainly confers the potential to induce attachment, colonization of the host, and the ability to bypass host defense mechanisms [11] (Figure 1). Failures in LPS biosynthesis cause cellular defects, resulting in envelope-responsive signal transduction controlled by RNA polymerase, extracytoplasmic E (RpoE) sigma factor, and two-component systems (TCSs). RpoE regulon members and specific TCSs (including their noncoding arm) regulate incorporation of nonstoichiometric LPS modifications, impacting on LPS heterogeneity and antibiotic resistance [16].

Experimentally, LPS injections cause nonspecific and acute reactions that trigger systemic inflammation implicated in many common disease states encountered throughout life. Among these states are maternal immune activation (MIA) [17] and the pathogenesis of cardiovascular diseases, chronic kidney diseases, autoimmune diseases, cancer, depression, and neurodegenerative diseases [18,19]. Specifically, within the host system, LPS binds to the cluster of the differentiation 14 (CD14)/toll-like receptor 4 (TLR4)/myeloid differentiation factor 2 (MD2) receptor complex found in monocytes, dendritic cells, macrophages, and B cells. The subsequent response depends on the type of cell to which LPS is bound [20]. TLR4 activation triggers the biosynthesis of different inflammation mediators, such as tumor necrosis factor (TNF) and interleukins (IL-1, IL-6, IL-8, IL-18), which stimulate the release of prostaglandins and leukotrienes [11,18], eventually leading to inflammation and septic shock, which are the most significant features of endotoxemia [21,22]. Moreover, TLR4 activation triggers the production of costimulatory molecules necessary for adaptive immune response [23]. Inflammatory mediators appear central in driving behavioral modifications, as in the case of the behavioral responses to LPS, known as sickness behavior, which is the acute consequence of cytokine elevation [18,24]. TLR4 activation also leads to the release of histamine, causing vasodilation, and activates clotting factors that lead to problems such as thrombosis, acute disseminated intravascular coagulation, hemorrhage, and septic shock [11].

It is important to emphasize that there are many factors that determine the response of the nervous system to the exposure to neurotoxic substances such as LPS. Species, gender, genotype, age, nutritional status, body temperature, and the type of environment the individual is exposed to are key determinants [1].

### 1.2. Environmental Enrichment (EE)

Complex genetic backgrounds and epigenetic processes shape the basic structure and function of the brain throughout life. Various environmental stimuli (positive or negative) and their interaction with genes shape the structure of neural circuits at a level of complexity that far exceeds the content of genomic information [25]. Thus, whether they are adaptive or maladaptive, environmental factors are fundamental in relation to neuroplasticity [26]. 

Neuroplasticity accounts for the ability of the brain to change in both structure and function as a consequence of life experiences. Enhanced stimulations provided by the environment are able to create brain, cognitive, and neural reserves which allow an individual to cope better with environmental demands, also in presence of neural damage (reserve hypothesis [27,28,29,30]). 

The importance of environmental stimulations was advanced by Donald Hebb’s observations; Hebb showed that socially enriched rats performed better on problem-solving tests than their counterparts reared in standard housing [31]. This observation spawned the concept of environmental enrichment (EE), especially through the work of Rosenzweig and colleagues [32,33,34,35]. Rosenzweig further defined the concept by classifying EE as a combination of social stimulation and exposure to diverse inanimate objects [32,36].

A considerable number of studies have associated protection against cognitive decline with three lifestyle components in both early and adult life, namely, social, mental, and physical factors [37,38,39,40]. Results have showed that a rich social network has a protective effect in coping with cognitive decline [41]. Beneficial effects have been demonstrated for mental components (such as high educational level, high work complexity, and a large amount of mentally demanding activities) and for regular physical activity and healthy dietary and lifestyle patterns [39,42,43,44,45,46,47,48].

Thus, EE has been reported to result in a wide range of brain changes, notably structural and functional remodeling of synapses linked to improved learning and memory, increased regulation of neurogenesis, glycogenesis, angiogenesis, and neurotrophic factors, as well as modulation of neuroinflammation, inflammation, immune senescence, and DNA epigenetic modification [29,36,40,49,50,51,52,53].

Despite the clinical relevance of the reserve hypothesis, some limitations and difficulties have been reported with respect to its experimental support. In fact, in clinical studies, several disturbance variables (linked to individual genetic patterns and life experiences) render it difficult to compare human subjects in terms of the effects of well-defined factors [30,47,54]. A useful and controllable means to investigate the mechanisms through which environmental stimuli exert their beneficial effects has been provided by murine models. Indeed, social, mental, and physical factors identified as protective in humans may be paralleled by manipulating a number of factors in rodent housing conditions. The enrichment may be achieved by enhancing only one of the three involved domains or more than one of them in combination, thus obtaining complete experimental control of the manipulated variables. In addition, the manipulation can be directed to stimulate an individual sensory channel or multiple channels in combination [33,40,50,54].

Interactions between the individual and the environment occur from conception throughout life. In fact, during fetal development, in addition to the overwhelming impact of genetic control, environmental stimuli strongly influence the highly susceptible developing structures. Namely, the environment experienced by the pregnant mother (interestingly, even before fertilization) exerts substantial effects on the intrauterine milieu, affecting fetal development, as well as on herself [55,56,57,58,59]. In addition, in aged rodents, daily exposure to EE primarily stimulates neurogenesis by increasing the probability of neuronal survival and of the rapid acquisition of spatial information [60,61,62,63,64,65].

Based on the above, the purpose of this review is to describe the effects that EE paradigms can bring in counteracting LPS-induced neuroinflammation, throughout the lifespan, tested in preclinical murine models.

## 2. Methodology of the Literature Search

A methodical search of the literature was performed on PubMed and Scopus databases to identify articles focused on the interaction of two fields of interest: LPS, as inflammatory mediator, and exposure to EE. Only studies written in English and performed on rodent models were considered. Only articles published up to 4 October 2022 were reviewed.

The advanced search method for both databases was as follows: (environmental enrichment OR enriched environment) AND (LPS OR lipopolysaccharide) AND (mice OR mouse OR rat OR rats OR rodent*).

The PubMed search produced a total of 212 articles while the Scopus search produced a total of 37 results. There were 27 duplicate articles. After selection based on reading titles and abstracts, 34 publications were temporarily included. Of these, 12 publications were discarded after reading the full text because they did not meet our inclusion criteria, while the remaining 22 were included in the present review.

Inclusion criteria were:

1. As the population of interest, we selected rodents, both healthy and pathological models, with no sex or age restrictions.
2. As the intervention of interest, we selected multidimensional EE exposure of animals submitted to LPS treatment carried out in any period of life, before or after the exposure to EE. For models based on transgenerational and MIA models, studies on enriched mothers and LPS-injected pups, and vice versa, were also considered.
3. As the control group of interest, we selected animals submitted to LPS treatment and reared under standard laboratory conditions.
4. As outcomes of interest, we selected cognitive, emotional, motor, and social effects and related structural, physiological, or biochemical processes.

**Figure 2:**
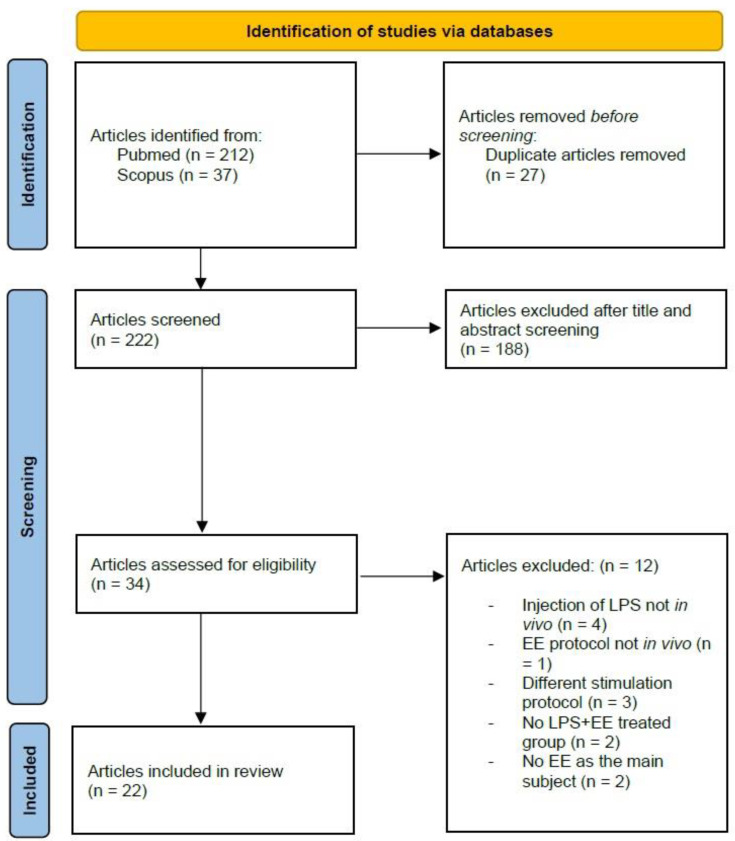
Search flow diagram based on PRIMA guidelines [66]. EE: environmental enrichment; LPS: lipopolysaccharide.

Figure 2 shows a detailed flow chart of the literature search, performed in accordance with the Preferred Reporting Items for Systematic Reviews and Meta-Analyses (PRISMA) [66] statement.

## 3. Beneficial Effects of Exposure to EE on Neuroinflammation Induced by LPS Injections

The evidence available in the literature on the topic is presented in three sections:

1. The first section reports the findings related to the beneficial effects of EE in case of prenatal neuroinflammation induced by LPS injections. The beneficial effects provided by exposure to EE for mother and offspring, respectively, are described.
2. The second section is devoted to the beneficial effects of EE in case of neuroinflammation induced by LPS injections in early age.
3. Finally, the third section includes the beneficial effects of EE in case of neuroinflammation induced by LPS injections in adult age.

### 3.1. Beneficial Effects of Exposure to EE on Mothers and Offspring in Case of Gestational Neuroinflammation Induced by LPS Injection

The early stages of development are very sensitive to the environment. Events that are stressful for the developing brain can have dramatic consequences on neurodevelopment [67]. 

Maternal health during pregnancy plays an important role in determining health and disease risks in the offspring. MIA hypothesis proposes that inflammatory perturbations in utero may affect fetal neurodevelopment [17] and increase the probability of developing neuropsychiatric, neurological [68], and neurodevelopmental disorders, such as autism [69] and schizophrenia [70,71]. Epidemiological studies in humans support the hypothesis that several maternal inflammatory factors, including obesity [72], psychosocial stress [17], autoimmune conditions [73], asthma and allergic conditions [74], and maternal viral infections [75,76,77] may be responsible for MIA [78]. MIA is mediated by the activation of inflammatory pathways that lead to increased levels of cytokines and chemokines, which reach the fetus by crossing the placental and blood–brain barrier [78,79,80]. Preclinical models of MIA created by using pathogens of Gram-negative bacteria, such as LPS and viruses, display anatomical and neurochemical changes corresponding to human disorders.

In contrast to the harmful effects of MIA, a healthy lifestyle is important not only in preventing possible diseases, but also to better enjoy life [81]. A healthy lifestyle is particularly important in pregnancy because the maternal lifestyle affects the mother’s health, pregnancy outcome, and fetus. For example, it is known that physical activity in leisure time [82,83] and antistress treatments (such as psychological accompaniment, midwife-led continuity of care, or relaxation therapy) [84] improve maternal health and reduce gestational disease. Preclinical studies are particularly indicative of the involvement of MIA in subsequent social and cognitive deficits of the offspring.

In this framework, we present a broad overview of the various possible interactions between EE and LPS-related neurotoxicity in the context of prenatal immune activation. We selected studies that examined the effects of exposure to enhanced environmental stimulations on mothers and/or their offspring, in the presence of LPS-induced pro-inflammatory stimulation during pregnancy. Details on the studies cited in this section are provided in Table 1 (study designs) and Table 2 (effects of the exposure to EE).

#### 3.1.1. Beneficial Effects of the Mother’s Exposure to EE in Case of Gestational Inflammation Induced by LPS Injection

Several studies have shown that interventions aimed at reducing maternal stress and anxiety have positive effects on pregnancy complications such as preeclampsia, excess gestational weight, gestational diabetes, and preterm birth [96,97,98]. In two recent animal studies, Schander and colleagues [84,85] examined the possible beneficial effects of exposure to an enriched environment (defined as a biological stimulus of the sensory pathway—meaningful but not invasive—combined with voluntary physical activity) on the triggering of premature labor and on the dangerous consequences of immune activation on pregnancy. The experimental design of both studies was based on the housing of 6-week-old female mice in an enriched environment for 6 weeks before mating and during the following 15 days of gestation. On gestational day (G) 15, the females were intraperitoneally (i.p.) injected with LPS to induce immunological challenge. One group of animals was followed to assess the rate of preterm birth, while another was euthanized to collect tissue. The exposure to EE prevented the preterm birth rate by 40% and modulated the maternal white blood cell count and the response to LPS. Furthermore, the findings indicated that EE modulated the maternal metabolism and immune response to systemic administration of LPS, producing an anti-inflammatory environment that contributes to the maintenance of pregnancy and the protection of the offspring’s development [85]. In utero, EE was found to reduce the expression of the LPS receptor—TLR4—and of the LPS coactivator protein CD14, inhibiting Prostaglandin E2 (PGE2) and Prostaglandin F2α (PGF2α) release and nitric oxide synthase (NOS) activity. Moreover, EE conserved cervical function by inhibiting cervical ripening events [84]. In addition, EE modulated the immune response in both peripheral blood and amniotic fluid. During lactation, EE prevented LPS-induced delay in physical landmarks analyzed to assess offspring development [85]. These results suggest that EE is able to modulate the mother’s immune response to systemic administration of LPS and to protect the offspring.

Gestational inflammation has been shown to accelerate age-related memory deterioration in mother mice [99]. In this regard, Zhuang and colleagues [86] investigated the protective effects of exposure to EE during the gestational phase. Female mice (about 2 months of age), housed in standard cages, received a daily i.p. injection of LPS during G 15–17 and then were left undisturbed to continue pregnancy and lactation. After weaning, a number of dams were exposed to EE until 6 or 18 months of age. The results showed that all 18-month-old mice experienced a noticeable decrease in mRNA/protein levels related to mitochondrial dynamics, biogenesis, and mitophagy in the hippocampus compared to 6-month-old mice. Regardless of age (6 or 18 months), mice injected with LPS during gestation performed worse in spatial learning and memory tests than control mice. The exposure to EE until 6 months of age failed to alleviate such a spatial learning impairment, whereas the exposure to EE until 18 months of age improved spatial learning performance compared to the LPS-only mice group, bringing the performance up to the levels of the control group. These results indicate that long-term exposure to EE modulates immune responses and protects against impairment of spatial cognition mediated by hippocampal mitochondrial quality control (MQC), thus demonstrating the beneficial effects of a sustained healthy lifestyle in counteracting the accelerated age-related deterioration in mother mice that experienced inflammation during pregnancy [86]. 

#### 3.1.2. Beneficial Effects of the Offspring’s Exposure of EE in Case of Gestational Inflammation Induced by LPS Injection

Prenatal inflammation causes chronic cognitive deficits, including learning and memory problems in the offspring [100,101]. Housing in cognitively, socially, and/or physically stimulating environments could alleviate the consequences of prenatal inflammation [102,103,104].

Connors and colleagues showed that EE protects male rat offspring from the sex-specific hypothalamic–pituitary–adrenal (HPA) axis and social interaction disorders that follow early MIA. Female rats were housed for about 2 months (during mating and gestation) in enriched or standard environments. Offspring were exposed to the same rearing conditions for the first 40 days of life. On G 11, all mothers were i.p. injected with LPS to induce MIA. EE prevented increased hippocampal corticosterone levels, decreased expression of glucocorticoid receptors, and reduced social contact in the young male offspring of LPS-treated mothers, while it was unable to protect against either the spatial deficits imposed by MIA or the subsequent reduction in hippocampal glutamate [87].

Rajesh and colleagues [88,89] investigated, in rats prenatally (from G 14 until parturition) exposed to i.p. LPS-induced inflammation, the effects of 15 min a day of physical exercise (treadmill), along with 4 h a day of multidimensional EE, for 45 days (postnatal days (P) 15–60), in mitigating learning and memory disorders and improving changes in hippocampal dendritic morphology during youth. Exercise and EE during adolescence mitigated spatial learning and memory deficits in subjects exposed to prenatal inflammation [88]. Furthermore, being raised in an enriched environment with a treadmill improved the dendritic arborization of hippocampal CA3 neurons in adolescent rats prenatally exposed to LPS-induced inflammation [89].

It is known that the development and plasticity of the neuroendocrine system can be influenced by many factors and that adverse prenatal events can cause lasting changes in adulthood. Bakos and colleagues [90] showed that simulating maternal inflammation by repeated subcutaneous treatment with LPS during gestation failed to influence basal hormone levels in adult rat offspring but did have an impact on their growth and on brain dopamine levels in the nucleus accumbens. Postnatal EE treatment eliminated the consequences of prenatal stress, leading to balanced growth and reverting the dopamine level decrement [90].

Early life is a critical period of development, and experiences at this stage have been shown to have a long-term influence on developmental processes, including in adolescence and aging [105,106]. Kentner and colleagues [91] investigated EE’s ability to reverse behavioral and neural alterations induced by prenatal i.p. administration of LPS used as a model of schizophrenia. In this study, the enrichment condition included the exposure (from P 50 for 6 weeks) to stimulating or socially (colony nesting) enriched environments. Findings showed that prenatal inflammation reduced the expression of the excitatory amino acid transporter (EAAT)-2 gene in the prefrontal cortex (PFC), an effect that was rescued by objectual enrichment. Furthermore, MIA, occurring at G 11, was associated with a downregulation of the brain-derived neurotrophic factor (BDNF) and neurotrophic tyrosine kinase receptor type 2 genes in the hippocampus and PFC, attenuated by environmental complexity. Furthermore, the prenatal infection caused disruption of social and spatial discrimination, with only the latter remedied by the social enrichment [91].

More recently, studies based on MIA induced by daily i.p. LPS injections during G 15–17 showed that spatial learning and memory of pups were impaired until middle age [92,93], suggesting that embryonic inflammation may accelerate the cognitive impairment associated with brain aging. Mice about 8-weeks-old were exposed to EE from 2 months of age [92] or from weaning [93] until the end of the experiments, i.e., about 3 or 15 months of age. EE alleviated deficits of hippocampal synaptic plasticity (hippocampal expression of the RNA-binding protein Staufen), memory performance, and anxiety symptoms caused by embryonic inflammation [92]. High levels of activity-regulated cytoskeleton-associated protein (Arc) and synaptotagmin-1 (Syt1) genes were observed in middle-aged mice of LPS-injected dams. Once more, a beneficial effect of EE was revealed [93]. Moreover, the exposure to EE from adolescence facilitated spatial learning and memory by downregulating the expression of Arc and Syt1 in the hippocampus [93].

Zhao and colleagues [94] demonstrated that a single i.p. LPS injection during mid-gestation (G 15) abolished spatial discrimination ability in female rats. Post-pubertal EE (P 50–P 92) rescued behavioral deficits in spatial discrimination ability and modulated elevations in plasma corticosterone caused by MIA. Moreover, within the PFC, hippocampus, amygdala, and hypothalamus, there was a downregulation of markers associated with the HPA axis and glutamate signaling pathways. Finally, MIA and EE altered the mRNA expression of several genes associated with resilience and synaptic plasticity [94].

Anxiety-related disorders and cognitive deficits are common in patients with epilepsy. Previous studies have shown that MIA makes children more vulnerable to neurological disorders later in life [107] and EE has been suggested to improve seizures, anxiety, and cognitive impairment in animal models [108,109,110,111]. In a recent study, Zeraati and colleagues [95] found that male offspring born to mothers i.p.-treated with LPS were more vulnerable to induction of seizures in adulthood compared to LPS female or saline male offspring. In any case, about 3 months of EE reversed the effects of prenatal immune activation on behavioral and inflammatory parameters in the epileptic offspring. Specifically, EE improved memory functions and reduced anxiety-like behavior [95]. These results demonstrate the significant association between an enriched and stimulating environment and reduced neuropsychiatric comorbidities in epileptic offspring.

### 3.2. Beneficial Effects of Exposure to EE in Case of Early Postnatal Neuroinflammation Induced by LPS Injection

Early childhood inflammation is commonly associated with changes in social and spatial memory and neurodevelopmental problems [112,113,114,115]. Environmental stimulation during early postnatal periods accelerates neurodevelopment, promotes neurogenesis and cell proliferation, modifies sensory and motor circuits, increases levels of neurotrophic and growth factors, improves memory, reduces stress levels, and increases dendritic spine density [116,117,118,119].

In this framework, we selected studies examining the effects of exposure to EE in the presence of LPS injection during early postnatal age. Details on the studies cited in this section are provided in Table 3 (study designs) and Table 4 (effects of the exposure to EE).

MacRae and colleagues [120] addressed the consequences of neural and behavioral reprogramming caused by neonatal inflammation in different environmental conditions in adolescence and adulthood. To this aim, female rats were housed under EE or standard conditions from breeding to offspring weaning. Subsequently, their offspring were exposed to the same rearing conditions for 90 days after birth. Pups were subjected to early postnatal inflammation with two i.p. LPS injections on P 3 and P 5. Social behavior and spatial memory were evaluated in males and females, at adolescent age (P 40) and in adulthood (P 70). Standard-housed, LPS-treated male and female rats, but not the EE-exposed ones, had disrupted juvenile social interactions (P 40) which were remitted by maturity (P 70). Furthermore, in adolescence, LPS-treated males had reduced glutathione levels in the PFC, which were normalized in EE-exposed peers and in adult animals. In contrast, LPS-treated male rats showed intact spatial memory as adolescents, which was impaired in old age. Despite the known benefits of EE, especially with regard to cognition [34], no protection against spatial damage in advanced age was described. EE offered some protection against the consequences of inflammation on juvenile social behavior and fully prevented reduced glutathione levels in the juvenile PFC. To ensure that LPS treatment on the litter did not affect maternal behavior, maternal care and anxious behaviors of the mothers were monitored. Interestingly, enriched mothers started retrieving pups earlier and reunited the entire litter more quickly than standard-housed rats during the home cage retrieval task. Neonatal inflammation did not alter the overall passive mothers’ behavior, although standard mothers exhibited specific differences in caring for pups (i.e., increased frequency of low crouch nursing and duration of time on the nest). These findings suggest that LPS-induced reprogramming is expressed in a time-, gender-, and environment-dependent manner and that an enriched environmental context might offer neuroprotection, especially in the early stages of life [120]. 

Wu and colleagues [121] hypothesized that early immune challenge induced activation of microglia, contributing to synaptic and cognitive disturbances in adolescent mice. On P 10, offspring received a single i.p. LPS injection and, subsequently, pups were exposed to EE for 4 h per day during P 10–38. After that, behavioral, biological, and structural analyses were carried out. Results demonstrated that early life LPS injection induced a working memory impairment rescued by EE. In association, EE prevented the activation of microglia, the excessive engorgement of inhibitory synapses, and the decreased number of perisomatic points on both inhibitory parvalbumin interneurons and excitatory neurons of the hippocampus and medial PFC. Finally, EE rescued the LPS-induced loss of dendritic spines in the CA1 of the hippocampus in adolescent mice. 

Pavlova and colleagues evaluated the effects of approximately 2 months (starting from the age of 45 days) of housing under different conditions—standard rearing, social isolation, or EE (20 min per day)—on the anxiety–depressive behavior of adult rats previously subjected to early pro-inflammatory stress, i.e., i.p. LPS injection on P 3 and P 5. Social isolation led to increased anxiety and risk assessment behavior, with some reductions in movement and exploratory activity mainly in LPS-treated rats. In association, no beneficial effects of EE were found on anxiety–depressive symptoms, which even appeared to increase in enriched LPS-treated female rats [122].

### 3.3. Beneficial Effects of Exposure to EE in case of Neuroinflammation Induced in Adulthood by LPS Injection

As for the developmental phases that follow early postnatal age, articles that used enrichment protocols in counteracting systemic inflammation induced by LPS injection in rodents during adulthood were selected; details on the studies cited in this section are provided in Table 5 (study designs) and Table 6 (effects of the exposure to EE).

Mlynarik and colleagues evaluated whether the stress response induced by repeated immune challenges could be altered by the type of environment the animals were exposed to. Specifically, they examined the effects of 5 weeks of different preventive housing conditions in 2-month-old male rats exposed to immune stress stimulation (increasing i.p. doses of LPS for 5 consecutive days). EE protected rats from the increased plasma and adrenal corticosterone levels and the transient decrease in body weight induced by LPS. Furthermore, EE abolished corticosterone response to mild repeated immune challenge, indicating a better ability of enriched rats to cope with stress, thus elucidating the possibility that differences in housing conditions may modify the stress response [123]. Williamson and colleagues examined the impact of preventive EE on neurogenesis, gliogenesis, neurotrophin, cytokine, and chemokine expressions, and glial alterations within the hippocampus and cortex of 2-month-old male rats housed 12 h/day in an enriched environment (or in a standard home cage) for 7 weeks and subsequently i.p. injected with LPS. Cytokine and chemokine levels were also assessed within the periphery. A markedly reduced pro-inflammatory phenotype within the brains of EE rats compared to that of LPS-treated ones was observed, evidenced by the selective attenuation of a subset of cytokines and chemokines following peripheral LPS administration. This attenuation was restricted to the hippocampus and coincident with increased expression of the glial antigens ionized calcium-binding adaptor molecule 1 (Iba1) and glial fibrillary acidic protein (GFAP). Surprisingly, however, these changes were independent from increased neurogenesis. Specifically, expression of the chemokines Ccl2, Ccl3, and Cxcl2, several members of the TNF family, and the pro-inflammatory cytokine IL-1β, were all significantly decreased following LPS administration in EE rats compared to controls. EE did not impact the inflammatory response to LPS in the cortex. Moreover, EE significantly increased both astrocyte and microglia antigen expression within the dentate gyrus. Measures of neurogenesis were not impacted by EE although EE significantly increased hippocampal BDNF mRNA. These findings demonstrate the importance of environmental factors on the function of the immune system, specifically within the brain [124]. On the whole, the two studies [123,124] demonstrate how an enriched and stimulating environment can provide protection against immune challenges, leading to different responses to stress and profoundly modifying immune response and neural function.

Other studies have been devoted to investigating the therapeutic potential of exposure to EE occurring after LPS administration, used as model of a number of pathological conditions involving neuroinflammation.

Acute systemic inflammation secondary to infection and tissue injury has been reported to induce various cognitive complications, such as delirium and postoperative cognitive dysfunction [130,131,132]. Cognitive disturbances associated with inflammation occur commonly, but not exclusively, in elderly patients and are associated with a significant increase in length of hospitalization, need for institutionalization, and long-term mortality [133,134]. Although the pathogenic mechanisms of inflammation-induced cognitive dysfunction remain elusive, pro-inflammatory cytokines, such as TNF-a and IL-1b, are thought to play an important role in such a process [135,136]. Kawano and colleagues explored the potential effects of 7-day multisensory rehabilitation on cognitive dysfunction following LPS-induced systemic inflammation. Following i.p. LPS injection, 25-month-old rats showed reduced memory performance and augmented hippocampal levels of both TNF-α and IL-1β in comparison to control animals. Conversely, in the group treated with LPS and multisensory rehabilitation, neither cognitive impairment nor increase in hippocampal TNF-α and IL-1β levels were found [125].

Neuroinflammation is a primary pathophysiological condition that is associated with the cognitive impairment that characterizes neurodegenerative diseases such as Alzheimer’s disease, Parkinson’s disease, multiple sclerosis, Huntington’s disease, amyotrophic lateral sclerosis, fronto-temporal dementia, and tauopathies [137,138]. Many reports indicated that neuroinflammation is related to the activation of microglia and is also accompanied by the excessive release of pro-inflammatory and neurotoxic mediators [139,140]. Inflammatory agents, such as IL-6, have the ability to cross the blood–brain barrier [141] and can inhibit the expression of BDNF genes, which are essential for hippocampal-dependent learning and memory [142]. A growing number of epidemiological and translational studies indicated that systemic inflammation may promote neurodegenerative diseases [143]. In this framework, Keymoradzadeh and colleagues [126,127] focused on the effect of EE on learning and memory impairments following LPS-induced neuroinflammation. In 7-week-old rats, i.p. LPS injections at days 1, 3, 5, and 7 caused memory impairment. Two weeks after the first injection, groups were transferred to standard or enriched environments for three weeks. Results indicated that LPS significantly impaired spatial learning and memory [126] and passive avoidance memory [127], and EE significantly improved these impairments. The preservation of cognitive function was associated with decreased IL-1β level and increased IL-10 level in the hippocampus in respect to the levels observed in standard-reared LPS-animals [126]. Moreover, EE increased the BDNF level and reduced the IL-6 level in the hippocampus, by acting in reverse direction to LPS administration [127].

Sepsis is a rare complication of infection, and its consequences can be very serious and potentially fatal. It consists of a disproportionate inflammatory response to a generalized infection that damages tissues and organs and impairs their function. Sepsis is a major clinical challenge associated with multi-organ dysfunction, including sepsis-associated encephalopathy (SAE) [130,144,145]. SAE is characterized by increased risk for the development of mental, cognitive, and physical impairments that may persist for months or years [130,144,145]. Ji and colleagues [128] tested the hypothesis that behavioral outcomes of i.p. LPS administration to 3–4-month-old male mice were alleviated by a 4-week exposure to EE. Results partially confirm this proposal. While no effects of EE on anxious behavior and on object recognition memory were observed, EE reversed the impairment in contextual fear conditioning response. Regarding inflammatory responses, only hippocampal IL-6 levels were increased in the LPS group and rescued in the EE group. Furthermore, EE did not reverse the LPS-induced decrease in hippocampal neurogenesis, neuronal dendritic spine number, and BDNF expression [128].

Optic neuritis is an inflammatory, demyelinating, neurodegenerative, and presently untreatable condition of the optic nerve which may induce blindness. LPS injection into the optic nerve represents an experimental model of primary optic neuritis [146]. Results reported by Aranda and colleagues [129] on 2-month-old male rats with LPS-induced optic neuritis, exposed to a standard environment or EE for different intervals (21, 17, 14, or 3 days) indicate that EE preserved visual functions, reduced neuroinflammation of the optic nerve, ameliorated optic neuritis symptoms, and reverted microglial/macrophage reactivity and astrocytosis [129].

## 4. Discussion

Neuroinflammation is related to the activation of the brain-resident immune cells and microglia. It is also accompanied by the excessive release of pro-inflammatory and neurotoxic mediators [139,140]. Both in vivo and in vitro studies have shown that LPS can induce systemic inflammation and damaging effects on the brain through the upregulation of several pro-inflammatory mediators, including nitric oxide species, PGE2, cyclooxygenase-2 and pro-inflammatory cytokines such as IL-1, IL-6 and TNF-α [147,148,149]. Moreover, LPS injection is known to be associated with hippocampal neuroinflammatory processes [150] and memory impairment in several animal models of neuroinflammatory diseases [151,152]. Environmental risk factors, such as poverty, stressful urban environments, and negative social interactions (such as bullying and abuse in childhood or adolescence) can act synergistically to increase susceptibility to progressive neurodevelopmental disorders [153]. Nowadays, it is generally accepted that no effective therapy has been developed for the treatment of chronic inflammation damages and research focus has shifted towards more promising preventive approaches. Numerous studies have observed that health can be improved by the exposure to stimulating and enriched environments; cognitive function and synaptic transmission have been shown to be affected by lifestyle changes [27,154,155]. 

In the present review, we investigated the importance of an enriched and stimulating environment in counteracting the damage caused by LPS-dependent neurotoxicity. On the whole, we found that exposure to EE is able to exert sex- and age-dependent neuroprotective and therapeutic effects. The studies included in the review indicate that such effects are present both in rodents with generic LPS-induced neuroinflammation [84,85,86,87,88,89,90,91,92,93,94,95,120,121,122,123,124,125,126,127] and in pathological models where LPS injection is used to develop a specific condition [128,129]. Evidence is available throughout life, namely, on LPS administration in the prenatal period (to the mothers), in early age, and throughout adult life until old age.

As for prenatal LPS administration, animal models of MIA display impairment in behavioral and cognitive functioning of offspring [17] and mothers [105]. Of particular interest are the studies (*n* = 3) that address the role of the exposure of the mothers to a healthy, enriched and stimulating environment in countering the damage of immune activation resulting from gestational LPS injection. We found only one study focused on the direct consequences of neurotoxic damage on mothers and the role of EE in countering such damage [86]. Two other studies focused on the protective role of EE in reducing gestational problems and premature birth [84,85]. In these studies, Schander and colleagues show that EE modulates maternal metabolism and produces an anti-inflammatory environment that contributes to the maintenance of pregnancy. In the uterus, the expression of the LPS receptor, TLR4, and its coactivator protein, CD14, is reduced, thus preventing the LPS-dependent increased release of PGE2, PGF2α, and NOS. In cervical tissue, EE preserves cervical function, modulates maternal white blood cell counts, and increases IL-10 and B cell levels in amniotic fluid [84]. In association, EE prevents the negative influence of intrauterine exposure to an inflammatory environment on the offspring’s development during lactation [85]. Thus, on the whole, evidence is provided for the beneficial effects of the exposure of mothers to an enriched environment in cases of gestational inflammation; such effects support healthy gestation as well as pup birth and early development. However, since studies on this topic are still scarce, further studies could better characterize the potentiality of EE in respect to this specific target.

The remaining studies based on the use of an MIA model (*n* = 9) evaluate the effects of direct exposure to EE of offspring after birth [87,88,89,90,91,92,93,94,95]. Connors and colleagues [87] demonstrated that postnatal exposure to EE, when preceded by the exposure of the mothers to EE, mitigated the detrimental effects of MIA by protecting offspring against the sex-specific HPA axis and social interaction disruptions. Alternatively, EE failed to protect against the spatial deficits imposed by MIA [87]. A number of other studies evaluated the therapeutic action of exposure to EE only after the birth of the animals submitted to prenatal LPS administration. The findings by Bakos and colleagues [90] demonstrated that simulation of maternal inflammation by repeated LPS treatment failed to influence baseline hormone levels in adult offspring but had an impact on their growth, behavior, and brain dopamine levels. Surprisingly, enriched animals showed increased corticosterone levels and enlarged adrenal glands [90]. It appears that LPS may act on each tissue that makes up the HPA axis. A pituitary-independent effect of LPS on the adrenal gland with increased corticosterone secretion has also been demonstrated. It has been shown that LPS inhibits ACTH-stimulated corticosterone secretion in cultured rat zona glomerulosa cells [156]. MIA has been also identified as a significant risk factor for several neurodevelopmental disorders. Kentner and colleagues [91] showed that gestational LPS injection induces the downregulation of genes critical for synaptic transmission and plasticity, which can underlie the pathogenesis of neurodevelopmental disorders such as schizophrenia and autism; such an effect can be restored in an experience-dependent way. Furthermore, immune activation in the mothers induced a deficit of social and spatial discrimination in the offspring, but only the latter may be remedied through enriched experience [91]. In a model of epilepsy, MIA worsened seizures in offspring in a sex-dependent manner and increased anxiety and cognitive impairment. EE reversed such effects by improving cognitive performance and anxiety. Furthermore, epileptic mice exposed to EE showed a significant reduction in hippocampal TNF-α and IL-10 [95]. Few studies investigated how embryonic inflammation affects cognitive abilities and neurobiological parameters during aging and how the psychosocial environment affects inflammation-induced remote cognitive impairment. Wu and colleagues [92] investigated whether the environment (stressful or enriched) can induce changes in the RNA-binding protein Staufen hippocampal expression and whether these changes are related to cognitive impairment induced by prenatal inflammation. The results show worse cognitive performance in older than young mice and greater expression of Staufen, indicating that the enriched environment would mitigate and the stressful environment would accelerate these effects [92]. Zhang and colleagues also confirmed worse cognitive performance in older rather than younger mice subjected to MIA, and found elevated levels of Arc and Syt1 gene expression compared to control mice. An LPS-exposed and EE-treated group showed better cognition and lower levels of Arc and Syt1 protein and mRNA than LPS-exposed mice of the same age. However, EE mitigated, but did not counter, the effects of prenatal inflammation on cognitive and synaptic proteins. Finally, better cognitive performance and ameliorated neuronal dendritic arborization are observed when the exposure to prenatal inflammation is contrasted by an enriched environment combined with physical activity [88,89]. Overall, these studies demonstrated the beneficial long-term effects of EE treatment at functional and structural levels. However, some conflicting findings indicate that EE is not able to rescue the totality of the damage induced by prenatal inflammation. Studies devoted to deepening the investigation of this topic are required to clarify such inconsistencies.

As reported by a handful of studies (*n* = 3), the exposure to inflammation in early life results in time-dependent changes in brain and behavior. EE fully prevents the reduction of glutathione levels in the PFC of juvenile males [120] and reverses LPS-induced microglial activation in the hippocampus [121]. With respect to the functional aspects, in the case of early LPS administration, beneficial effects of the exposure to EE after LPS administration are present following the combined exposure of the mothers in prenatal phase and the pups after birth [120], even when only the pups are exposed to EE [121]. The beneficial effects involve working memory performance [121] and social behavior [120]. However, as for depressive-like or anxious behaviors, contrasting results are reported. Pavlova and colleagues [122] found that early postnatal administration of LPS makes females susceptible to any environmental change, be it depriving or improving. Overall, despite the evidence of the beneficial effects of exposure to EE for animals submitted to early postnatal LPS administration, even on this topic, the studies are still too scarce to provide a clear characterization of the potentiality of EE in affecting the effects of early inflammation.

Finally, several studies analyzed the effects of the exposure to EE on animals submitted to LPS-induced inflammation in adulthood (*n* = 7). Overall, such studies support EE’s beneficial effects on the consequences of neurotoxic exposure to LPS in the later ages of life. This is true when the exposure to EE occurs both before [123,124] and after [125,126,127,128,129] LPS injection, supporting the neuroprotective and therapeutic action of EE. However, as for EE’s effects on cognitive function, we found evidence only in the studies based on the exposure to EE after LPS administration. EE improves LPS-dependent cognitive impairment in learning and memory performances [125,126,127,128,129]. Conversely, no effect on anxious behaviors were described [128]. EE leads to a better response to stress by abolishing the response of corticosterone to mild repeated immune challenge [123], although LPS does not influence the basal corticosterone levels [87,90,120,157]. Consequently, it would be important to better investigate the role of EE in controlling rodent stress response in cases of immune challenge. The studies on adulthood also demonstrate that EE impacts neuroinflammation and plasticity markers. In particular, EE decreases IL-6 and increases TNF-α, IL-10, IL-1β, and BDNF levels in the hippocampus [125,126,127], but not in the PFC [128]. However, in respect to the molecular and structural basis of EE’s beneficial effects, contrasting results were found. Indeed, Ji et al. [128] report that EE did not reverse the LPS-induced decrease in hippocampal neurogenesis, neuronal dendritic spine number, and BDNF expression [128]. Thus, EE may reverse the damage induced by neurotoxicity but its beneficial effects may need longer and specific times [128,158,159,160,161]. It is noteworthy that EE’s beneficial effects (in neuroinflammatory molecular processes and memory performance) are also detected when inflammation occurs in aging (24–25 months) and the exposure to EE is short term (7 days) [125]. Thus, it is possible to affirm that available evidence strongly supports the beneficial effects of exposure to EE in counteracting the damage induced by adult exposure to LPS. Further studies are requested to deepen our understanding of aspects that are still unclear.

Overall, the present review confirms the hypothesis that EE has restorative and neuroprotective effects on LPS-induced neuroinflammation and that beneficial effects can occur regardless of age. However, exposure to neurotoxic agents, combined or not with stimulating environments, has different consequences on biochemical, morphological, and cognitive/behavioral aspects in relation to several factors (sex, age, duration of the exposure, LPS dosage, etc.).

## 5. Conclusions

**Figure 3:**
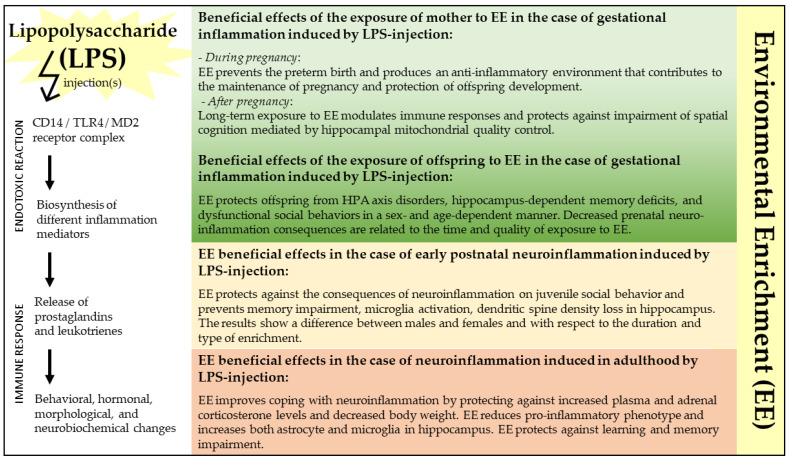
The figure illustrates the effects of exposure to environmental enrichment (EE) in counteracting lipopolysaccharide (LPS)-induced neurotoxicity across the lifespan. CD14: cluster of differentiation 14; HPA: hypothalamic–pituitary–adrenal; MD2: myeloid differentiation factor 2 (MD2); TLR4: toll-like receptor 4.

EE represents a powerful means to enhance brain plasticity mechanisms (e.g., synaptogenesis, neurogenesis, angiogenesis, molecular changes, etc.) and improve behavioral function in normal and pathological individuals. As summarized in Figure 3, the present review supports the use of EE protocols to counteract LPS-induced neurotoxicity, as both a protective and therapeutic factor. The analyzed studies demonstrate that EE’s effects extend across the lifespan, from prenatal to adult exposure. Although further experiments are needed to fully understand the mechanisms underlying EE’s beneficial actions and to study which aspects of EE (exercise, socialization, cognitive stimulation) represent the critical ingredients to enhance brain plasticity, it is possible to propose that an increasingly efficient and tuned use of enrichment protocols will allow the management of neuroinflammatory damage in clinical settings.

## Figures and Tables

**Table 1:** Prenatal inflammation—study designs.

	References	Species	Environmental Enrichment		LPS Injection(s)
		(Age and Timing of Evaluations)	Type of Enrichment	Subjects and Duration	(Time and Dose)
**EE on dams**	**Schander et al., 2020 [84]**	BALB/c mice Dams: 6 weeks (Effects on: mothers (pregnancy))	Large cage (640 × 420 × 200 mm) with running wheels and objects (toys, tunnels, shelters, and stairs) of different shapes, textures, and colors changed once a week. Every 3–4 days, one object removed, moved, or added. Ten animals per cage.	*On dams:* EE for 6 weeks; mating (regular cages); EE until G15.	On dams: G15, two i.p. injections of LPS (1°—0.13 mg/kg; 2°—0.39 mg/kg). (LPS after EE protocol)
	**Schander et al., 2021 [85]**	BALB/c mice Dams: 6 weeks (Effects on: mothers (pregnancy); offspring (P 1–P 22)	Large cage (640 × 420 × 200 mm) with running wheels and objects (toys, tunnels, shelters and stairs) of different shapes, textures, and colors changed once a week. Every 3–4 days, one object removed, moved or added. Ten animals per cage.	*On dams:* EE for 6 weeks; mating (regular cages); EE until G15.	On dams: G15, two i.p. injections of LPS (1°—0.13 mg/kg; 2°—0.39 mg/kg). (LPS after EE protocol)
	**Zhuang et al., 2021 [86]**	CD-1 mice Dams: 7/8 weeks (Effects on: mothers (after pregnancy))	Large cage (52 × 40 × 20 cm^3^) with toys (running wheels, tunnels, poplar wood block toys, and rings). Ten to fifteen animals per cage.	*On dams:* EE from weaning of pups until about 6 or 18 months of age of the mothers.	On dams: G15–17, daily i.p. injection of LPS (50 μg/kg). (LPS before EE protocol)
**EE on offspring**	**Connors et al., 2014 [87]**	Sprague–Dawley rats Dams: age not specified (Effects on: offspring (P 36–P 40))	Multi-level cage with toys changed two times weekly, tubes, chew bone, Nestlets©, and ramps. Pair-housed animals.	*On dams:* EE for about 2 months. *On pups:* EE from P 1 to P 40.	On dams: G11, i.p. injection of LPS (100 μg/kg). (LPS during EE protocol)
	**Rajesh et al., 2016 [88]**	Wistar rats Dams: 3 months (Effects on: male offspring (P 61–P 66))	EE: Large cage (120 × 100 × 100 cm) with plastic tunnels, raised platform with ladder, various size metal balls, toys, and steel swing. Objects were changed on alternate days. Treadmill running exercise: 1.5 m/min–10.9 m/min; 15 min/day. Number of animals per cage not specified.	*On pups:* EE (4 h/day) or/and treadmill from P 15 to P 60.	*On dams:* From G14 till parturition on alternate days i.p. injection of LPS (0.5 mg/Kg).
	**Rajesh et al., 2018 [89]**	Wistar rats Dams: 3 months (Effects on: male offspring (P 67))	EE: Large cage (120 × 100 × 100 cm) with plastic tunnels, raised platform with ladder, various size metal balls, toys, and steel swing. Objects were changed on alternate days. Treadmill running exercise: 1.5 m/min -10.9 m/min; 15 min/day. Number of animals per cage not specified.	*On pups:* EE (4 h/day) or/and treadmill from P 15 to P 60.	*On dams:* From G14 till parturition on alternate days i.p. injection of LPS (0.5 mg/Kg).
	**Bakos et al., 2004 [90]**	Wistar rats Dams: 8th day-pregnant—age not specified (Effects on: male offspring (~P 83))	Large cage (1 m × 0.5 m × 0.5 m) with bedding changed once a week, running wheels, wooden swings, plastic tubes, and beams changed three times a week. Occasionally added pieces of food (nuts, apples). Six animals per cage.	*On pups:* EE after weaning, for 2 months.	*On dams:* G15–19, daily subcutaneous injection of LPS in increasing doses of 20, 20, 40, 40, 80 μg/kg/mL.
	**Kentner et al., 2016 [91]**	Sprague-Dawley rats Dams: 12th day- pregnant—age not specified (Effects on: male offspring (~P 92)).	Communal nesting: larger-style one-level cage with tube, chew bone, and Nestlets©. Four animals per cage. EE: multi-level cage with chew bone, Nestlets©, ramps, novel toys, and tubes. Toys and tubes changed twice a week. Four animals per cage.	*On pups:* EE from P 50, for 6 weeks.	*On dams:* G15 i.p. injection of LPS (100 μg/kg).
	**Wu et al., 2020 [92]**	CD-1 mice Dams: 8 weeks (Effects on: male offspring (3 months old; 15 months old))	Large cage with pipes, plastic running wheels, balls, and toys changed every week. Number of animals per cage not specified.	*On pups:* EE from 2 months of age until 3 or 15 months of age.	*On dams:* G15–17, daily i.p. injection of LPS (50 μg/kg).
	**Zhang et al., 2022 [93]**	CD-1 mice Dams: 6/8 weeks (Effects on: male offspring (3 months old; 15 months old))	Large cage (36 × 23 × 18 cm) with climbing ladders, running wheel, ball, plastic and wooden objects suspended from the cage top, paper, cardboard boxes, nesting material, and toys (changed every 1–2 days). Eight animals per cage.	*On pups:* EE after weaning until 3 or 15 months of age.	*On dams:* G15–17, daily i.p. injection of LPS (50 μg/kg).
	**Zhao et al., 2020 [94]**	Sprague-Dawley rats Dams: 12th day- pregnant—age not specified (Effects on: female offspring (~P 92))	Communal nesting: larger-style one-level cage with tube, chew bone, and Nestlets©. Four animals per cage. EE: multi-level cage with chew bone, Nestlets©, ramps, novel toys, and tubes. Toys and tubes changed twice a week. Four animals per cage.	*On pups:* EE from P 50, for 6 weeks.	*On dams:* G15 i.p. injection of LPS (100 μg/kg).
	**Zeraati et al., 2021 [95]**	NMRI mice Dams: 70–80 days (Effects on: offspring—(P 121–P 142))	Cage (58 × 38 × 20 cm) with one running wheel, two plastic shelters, four tunnels, and two cotton nestlets replaced once a week. Four animals per cage.	*On pups:* EE from P 21 to P 120.	*On dams:* G17, i.p. injection of LPS (300 μg/kg/100 μL).

EE: environmental enrichment; G: gestational day; i.p.: intraperitoneal; LPS: lipopolysaccharide; P: postnatal day.

**Table 2:** Main effects of environmental enrichment in case of prenatal inflammation.

	References	Main Effects of Environmental Enrichment	
		On Mothers	On Offspring
	**Schander et al., 2020** [84]	EE reduced - body weight gain in nonpregnant mice; - cholesterol and triglycerides serum levels in pregnant mice;- LPS-induced preterm birth rate and offspring perinatal death (by 40%); - expression of TLR4 and CD14 in the uterus of LPS-challenged mice. EE prevented - LPS-induced increase in corticosterone serum levels; - LPS-induced neutrophil infiltration into the cervix as well as metalloprotease activity in this tissue. EE induced molecular changes in uterus and cervix of LPS-induced preterm birth mice.	
	**Schander et al., 2021** [85]	EE modulated - white blood cell count and its response to systemic LPS administration; - amniotic fluid response to LPS administration, promoting a tolerogenic microenvironment.	EE prevented the negative influence of intrauterine exposure to an inflammatory environment on physical landmarks (pinnae detachment, lower incisors eruption, eye opening) of the offspring’s development during lactation.
	**Zhuang et al., 2021** [86]	Long-term exposure to EE - reduced spatial learning and memory impairment *(MWM)* in aged dams resulting from LPS-induced gestational inflammation; - improved dynamics in hippocampi of LPS-injected dams; - alleviated the accelerated changes in mitochondrial biogenesis and mitophagy resulting from gestational inflammation in aged mice.	
**EE on offspring / EE on dams**	**Connors et al., 2014** [87]		EE prevented - elevations in hippocampal corticosterone level; - decreased glucocorticoid receptor expression in hippocampus; - reductions in social contact *(SI)* in juvenile male rats treated with LPS in utero.
	**Rajesh et al., 2016** [88]		Rats of LPS + EE + exercise group showed a significant enhancement in learning and memory performance *(MWM)*, compared with other groups.
	**Rajesh et al., 2018** [89]		Rats of LPS + EE + exercise group showed a significant enhancement in dendritic arborization of CA3 hippocampal neurons, compared with other groups.
	**Bakos et al., 2004** [90]		EE reversed LPS-induced nucleus accumbens dopamine level decrement.
	**Kentner et al., 2016** [91]		EE prevented the LPS-induced reduction of the expression of the EAAT-2 gene in the PFC; EE mitigated LPS-induced downregulation of BDNF in the hippocampus and neurotrophic tyrosine kinase receptor type 2 genes in PFC; colony nesting mitigated LPS-induced spatial and object memory impairment (object-in-place test).
	**Wu et al., 2020** [92]		EE alleviated LPS-induced memory impairments *(MWM)* in middle-aged mice.
	**Zhang et al., 2022** [93]		EE improved learning and memory performance *(MWM)* deteriorated by LPS injection; EE reduced protein and mRNA levels of Arc and Syt1 genes in the hippocampus increased by LPS injection.
	**Zhao et al., 2020** [94]		EE attenuated - recognition memory (object-in-place test) deficits observed in MIA offspring; - social (SI) deficits observed in MIA offspring; - the elevation of plasma corticosterone in MIA offspring.
	**Zeraati et al., 2021** [95]		EE improved - anxiety-like behavior in LPS offspring treated with water or PTZ compared to control groups (open field; light–dark box); - spatial working memory in LPS offspring treated with PTZ compared to control groups *(Y-maze)*; - recognition ratio in LPS offspring treated with PTZ compared to control groups (novel object recognition test). EE decreased - Seizure scores; - TNF-α and IL-10 levels in the hippocampus of LPS offspring treated with PTZ compared to normal-PTZ LPS offspring.

Arc: activity-regulated cytoskeleton-associated protein; BDNF: brain-derived neurotrophic factor; CD14: cluster of differentiation 14; EE: environmental enrichment; EAAT-2: excitatory amino acid transporter; LPS: lipopolysaccharide; MWM: Morris water maze; PFC: prefrontal cortex; PTZ: pentylenetetrazol; SI: social interaction test; Syt1: synaptotagmin-1; TLR4: toll-like receptor 4.

**Table 3:** Early postnatal inflammation—study designs.

References	Species	Environmental Enrichment		LPS Injection(s)
	(Age and Timing of Evaluations)	Type of Enrichment	Subjects and Duration	(Time and Dose)
**MacRae et al., 2015** [120]	Sprague–Dawley rats Dams: age not specified Pups: newborn (Evaluation of effects in male and female offspring (P 40–P 90))	Large multi-level cage with toys and tubes changed twice weekly, chew bone, Nestlets©, and ramps. Four to six animals per cage.	*On dams:* EE was conducted from breeding to weaning of pups. *On pups:* EE was conducted from birth to the end of the experiment (P 1–P 90).	*On pups:* P 3 and P 5, 2 i.p. injections of LPS (50 μg/kg). (LPS administered when EE protocol is already ongoing)
**Wu et al., 2022** [121]	C57BL/6 mice. Pups: 10 days (Evaluation of effects in male offspring P 39–P 42)	Cage (60 × 32 × 38 cm) with running wheel, swing, platforms and toys. Objects were changed twice a week. Number of animals per cage not specified.	*On pups:* EE was conducted four hours per day during P 10–P 38.	*On pups:* P 10, i.p. injection of LPS (100 μg/kg). (LPS before EE protocol)
**Pavlova et al., 2022** [122]	Wistar rats Pups: newborn (Evaluation of effects in male and female offspring P 90–P 105)	Plastic box (51 × 40.5 × 30 cm) containing stairs, a running wheel, tubes, and materials for burying (sawdust, foam filler, paper, etc.). Four to six animals per cage.	*On pups:* EE was conducted for 20 min every other day, from the age of 45 days until the end of experiment (~3.5 months).	*On pups:* P 3 and P 5, 2 i.p. injection of LPS (dose of 50 μg/kg in a volume of 10 μL/g). (LPS before EE protocol)

EE: environmental enrichment; i.p.: intraperitoneal; LPS: lipopolysaccharide; P: postnatal day.

**Table 4:** Main effects of environmental enrichment in case of early postnatal inflammation.

References	Main Effects of Environmental Enrichment	
	On Mothers	On Offspring
**MacRae et al., 2015** [120]	EE dams started retrieving pups earlier and reunited the entire litter more quickly than standard-housed rats.	EE partially protected against the consequences of inflammation on juvenile social (social interaction test) behavior. EE prevented LPS-induced glutathione level reduction in the juvenile PFC.
**Wu et al., 2022** [121]		EE improved LPS-induced working memory impairment *(Y-maze)*. EE attenuated - LPS-induced microglial activation in the hippocampus and mPFC; - LPS-induced loss of dendritic spines in the hippocampus. EE reversed LPS-induced decrease in parvalbumin expression in the hippocampus and mPFC.
**Pavlova et al., 2022** [122]		EE increased anxiety (open field) and depression-like behavior (sucrose preference test) in female LPS group.

EE: environmental enrichment; LPS: lipopolysaccharide; mPFC: medial prefrontal cortex.

**Table 5:** Inflammation in adulthood—study designs.

References	Species	Environmental Enrichment	LPS Injection(s)
	(Age)	(Type and Duration)	(Time and Dose)
**Mlynarik et al., 2004** [123]	Male Wistar rats 2 months	Plexiglas box (1 × 0.5 × 0.5 m) with sawdust straw bedding, several platforms, swings, and a variety of miscellaneous objects (plastic boxes, tunnels, cartons, iron ladders, glass bottles, laboratory beakers, natural branches, water pools, spin wheels). The floor configuration was changed every second working day and aforementioned items were randomly put in or taken out at the same time. A small amount of extra food (e.g., pieces of apples or oranges, peanuts, dried bread, and curd) were occasionally served or hidden in the straw bedding. Ten animals per cage. EE was conducted for ~5 weeks.	Daily i.p. injection of LPS in increasing doses of 10, 10, 20, 20, and 40 μg/kg/mL for 5 consecutive days. (LPS after EE protocol)
**Williamson et al., 2012** [124]	Male Sprague-Dawley rats 60 days	Boxes (55.9 × 35.6 × 30.5 cm) contained quarter-inch corn-cob bedding, a running wheel, a PVC tube, and various small objects and toys. Pair-housed animals. EE was conducted for 7 weeks for 12 h per day.	i.p. injection of LPS (100 μg/kg). (LPS after EE protocol)
**Kawano et al., 2014** [125]	Male Wistar rats 24–25 months	Large cage equipped with plastic toys, a tunnel, a ladder, a platform, nesting material, and a running wheel. Items were routinely rearranged during the experimental period. The multi-modal sensory stimulations were conducted by a buzzer sound (167 Hz), a blinking light-emitting diode (LED) light (3 Hz), and vibration (60 dB) for 1 min using a multi-digital tuner three times daily during the active phase. Pair-housed animals. EE was conducted for 7 days.	i.p. injection of LPS (5 mg/kg). (LPS before EE protocol)
**Keymoradzadeh et al., 2020** [126]	Male Wistar rats 7 weeks	Large cage (96 × 49 × 38 cm) containing running wheels, a tunnel, a small compartment, stairs, and many other colorful objects (e.g., colorful plastic plates, wooden disks of varied colors and sizes, plastic cups, and hanging cubes). Objects were changed every day. Number of animals per cage not specified. EE was conducted for 21 days (3 weeks).	i.p. injection of LPS (1 mg/kg) days 1, 3, 5, and 7. (LPS before EE protocol)
**Keymoradzadehet al., 2022** [127]	Male Wistar rats 7 weeks	Large cage (96 × 49 × 38 cm) containing running wheels, a tunnel, a small compartment, stairs, and many other colorful objects (e.g., colorful plastic plates, wooden disks of varied colors and sizes, plastic cups, and hanging cubes). Objects were changed every day. Number of animals per cage not specified. EE was conducted for 21 days (3 weeks).	i.p. injection of LPS (1 mg/kg) days 1, 3, 5, and 7. (LPS before EE protocol)
**Ji et al., 2017** [128]	Male C57BL/6 mice 3-4 months	Large cage (60 cm × 35 cm × 20 cm) containing a small house, a running wheel for voluntary exercise, and four to five toys that were exchanged three times a week for new toys of different shape and colors. Four to six animals per cage. EE was conducted for 4 weeks.	i.p. injection of LPS (5 mg/kg). (LPS before EE protocol)
**Aranda et al., 2019** [129]	Male Wistar rats 2 months	Big cages (46.5 × 78 × 95 cm) containing 4 floors and several food hoppers, water bottles, running wheels, tubes, ramps, and differently shaped objects (balls, ropes, stones) repositioned once a day and fully substituted once a week. Six animals per cage. Animals were continuously exposed to standard enrichment or EE during different intervals (21, 17, 14, or 3 days).	An amount of 1 μL of 4.5 μg/μL LPS in pyrogen-free saline locally injected in the optic nerve. (LPS before EE protocol)

EE: environmental enrichment, i.p.: intraperitoneal; LPS: lipopolysaccharide; P: postnatal day.

**Table 6:** Main effects of environmental enrichment in case of inflammation in adulthood.

References	Main Effects of Environmental Enrichment
**Mlynarik et al., 2004** [123]	EE prevented - LPS-induced transient decrease in body weight; - LPS-induced increase in plasma and adrenal corticosterone levels.
**Williamson et al., 2012** [124]	EE increased - density of glial markers within the DG altered by LPS injection; - BDNF mRNA in the hippocampus decreased by LPS injection. EE attenuated hippocampal response to LPS for a subset of cytokines and chemokines.
**Kawano et al., 2014** [125]	Multisensory early rehabilitation environment reverted - LPS-induced memory impairment (novel object recognition test); - LPS-induced elevation of TNF-a and IL-1b levels in hippocampus.
**Keymoradzadeh et al., 2020** [126]	EE ameliorated LPS-induced spatial learning and memory impairment (Morris water maze). EE counteracted LPS-induced increase of hippocampal IL-1β levels. EE further augmented LPS-induced increase of the hippocampal IL-10 levels.
**Keymoradzadeh et al., 2022** [127]	EE counteracted LPS-induced memory impairment (passive avoidance test). EE counteracted LPS-induced increase of IL-6 levels and decrease of BDNF levels in hippocampus.
**Ji et al., 2017** [128]	EE reverted - LPS-induced hippocampal and non-hippocampal-dependent cognitive impairment (fear conditioning test); - LPS-induced increased hippocampal IL-6 expression.
**Aranda et al., 2019** [129]	EE rescued - LPS-induced decrement in pupil light reflex, visual evoked potentials, retinal anterograde transport, phosphorylated neurofilament immunoreactivity, myelination, axon and retinal ganglion cell number; - LPS-induced optic nerve oxidative damage and LPS-increased optic nerve inducible nitric oxide synthase, cyclooxygenase-2, and IL-1b and TNF-α mRNA. EE counteracted the microglial/macrophage reactivity and astrocytosis observed in LPS group.

AMPA GluR1: alpha-amino-3-hydroxy-5-methyl-4-isoxazole propionic acid subunit glutamate receptor 1; BDNF: brain-derived neurotrophic factor; DG: dentate gyrus; EE: environmental enrichment; IL: interleukin; LPS: lipopolysaccharide; TNF: tumor necrosis factor.

## Data Availability

Not applicable.
